# Aloe-Emodin Modifies Adipogenic Genes and the Expression of miR-143 and miR-155 in 3T3-L1 Cells

**DOI:** 10.3390/life16091552

**Published:** 2026-09-16

**Authors:** Carlos Uriel Torres-Estrella, Jose Luis Acosta-Rodríguez, Julio Jesús Garcia-Coste, Fausto Sánchez-Muñoz, Rodrigo Romero-Nava, Karla Aidee Aguayo-Cerón

**Affiliations:** 1 Laboratorio de Investigación en Genética de Enfermedades Metabólicas, Escuela Superior de Medicina, Instituto Politécnico Nacional, Mexico City 11340, Mexico; ctorrese1300@alumno.ipn.mx (C.U.T.-E.); jjcoste18@gmail.com (J.J.G.-C.); 2 Centro Interdisciplinario de Investigación para el Desarrollo Integral Regional, Boulevard Juan de Dios Bátiz Paredes, Guasave 81049, Mexico; jlacostar@ipn.mx; 3 Departamento de Fisiología, Instituto Nacional de Cardiología, Juan Badiano No. 1, Col. Sección XVI, Tlalpan, Mexico City 140080, Mexico; fausto22@yahoo.com

**Keywords:** adipogenesis, miR-143, miR-155, gene expression, Aloe-emodin

## Abstract

Adipogenesis is crucial for maintaining energy homeostasis, as it facilitates the differentiation of mesenchymal stem cells into adipocytes. This process is transcriptionally regulated by key factors including PPARγ and C/EBPβ, alongside FABP4 during lipogenesis. However, it is now well established that a significant portion of this regulation is also mediated by small non-coding RNAs (miRNAs) and long non-coding RNAs (lncRNAs), which post-transcriptionally modulate gene expression. This study aimed to identify key miRNAs involved in adipogenesis and to determine how Aloe-emodin, a molecule with potent anti-inflammatory properties known to modulate adipocyte differentiation, alters their expression. Using an integrative approach combining 3T3-L1 cell culture, RNA-seq, and qPCR analyses, this study demonstrates that Aloe-emodin can upregulate transcription factors (PPARγ and C/EBPβ) as well as miR-143 and miR-155, suggesting its potential as a modulator of adipogenic gene or miRNA expression in this exploratory setting.

## 1. Introduction

### 1.1. Aloe-Emodin and Its Properties

Aloe-emodin is an anthraquinone derived from *Aloe vera*, a plant belonging to the genus Aloe and the family Liliaceae, native to South Africa, but it has also been recognized as endemic to tropical and subtropical climates, including North America [1,2]. It is known that this anthraquinone exhibits potent laxative properties, but it also possesses a wide variety of pharmacological properties, such as anticancer, antiviral, antibacterial, neuroprotective, and anti-inflammatory activities, which have prompted numerous studies on its impact on different metabolic processes and diseases [2]. Among these, studies investigating its effects on various cancers, such as lung, breast, and gastric cancer, stand out [3,4,5,6]. Similarly, its effect on cell differentiation during the adipogenesis in mesenchymal cells has been evaluated, and found to inhibit this process [7].

### 1.2. Adipogenesis and Obesity

Adipogenesis is the process through which multipotent mesenchymal stem cells (MSCs) differentiate into adipocytes. This differentiation is regulated by environmental factors, the expression of specific genes and proteins, as well as by miRNAs and transcription factors [3,8,9]. Its primary function is to store energy as lipids [10], which constitute adipose tissue (AT), one of the most complex tissues in the human body. Adipogenesis comprises three well-defined stages. The first involves the commitment of MSCs to the adipocyte lineage, thanks to multiple signaling pathways, notably phosphoinositide 3-kinase (PI3K) and protein kinases (AKT), which are recruited by the insulin signaling pathway and the mechanistic target of rapamycin complex 1 (mTORC1), both involved in pre-adipocytes formation [11]. Similarly, the mitogen-activated protein kinase/extracellular signal-regulated kinase (MAPK/ERK) pathway is also highlighted [12,13] as it is activated through the insulin pathway. These pathways recruit transcription factors that translocate to the nucleus to promote lineage commitment, including Zinc Finger Factor 423 (ZFP423), p38 mitogen-activated protein kinase (p38), bone morphogenetic proteins 2 and 4 (BMP2/4), and cyclic adenosine monophosphate (cAMP) response element-binding protein (CREB) [14,15,16].

The second stage occurs almost simultaneously with mitotic clonal expansion (MCE). To achieve this, cells are arrested in the G1 phase. Populations that overcome this restriction re-enter the cell cycle for one or two additional rounds, leading way to the early phase of differentiation and the recruitment of transcription factors [17]. These include the peroxisome proliferator-activated receptor gamma (PPARγ), which enhances insulin signaling, a process necessary for adipogenesis [11,18]. PPARγ is a nuclear hormone receptor transcription factor involved in multiple processes, including energy metabolism, cell proliferation, and inflammation. It has been demonstrated that PPARγ is essential for adipogenesis in both in vivo and in vitro models [19]. During this stage, another regulatory transcription factor involved is the coactivator CCAAT/enhancer-binding protein alpha (C/EBPα), which can also induce adipogenesis in fibroblasts. However, for both PPARγ and C/EBPα to function, they must first be recruited by C/EBPβ and C/EBPδ, whose nuclear localization facilitates their binding to DNA, thereby promoting the activation and phosphorylation of PPARγ and C/EBPα. This cooperative recruitment of enzymes that assist in differentiation leads to the final stage, known as “terminal differentiation”, which aims to activate genes that promote the transition to the adipocyte phenotype [16]. This phase induces the activation of specific adipocyte genes including lipoprotein lipase (LPL), fatty acid synthase (FAS), fatty acid-binding protein 4 (FABP4), perilipin, adiponectin (ADIPOQ), and the glucose transporter 4 (GLUT4). Consequently, these proteins are considered markers of terminal differentiation and mature adipocytes [20,21]. This complex process promotes “tissue remodeling”, during which cells undergo rearrangement of the extracellular matrix, alongside vascular expansion, monocyte recruitment, and activation of pro-inflammatory macrophages by mature adipocytes. This fosters an inflammatory state mediated by proteins such as tumor necrosis factor-alpha (TNF-α), interleukin 6 (IL-6), and interleukin 1-beta (IL-1β), as well as increased reactive oxygen species (ROS) [22,23], all of which are key to the development of obesity, largely resulting from the intense activation of this differentiation process. Obesity is a risk factor for multiple chronic diseases, including hypertension, diabetes, cardiovascular diseases, and respiratory disorders, among others that compromise systemic metabolism [24]. Since obesity is a multifactorial disease, genetic and environmental factors, along with diet, are critical to understanding its development. Specifically, sugars, saturated fats, and processed foods increase caloric load, inevitably leading to an energy imbalance due to excessive caloric intake [25].

### 1.3. miRNAs and Their Interaction with Metabolism and Adipogenesis

MicroRNAs (miRNAs) are small non-coding RNA molecules approximately 22 nucleotides in length that play a critical role in the post-transcriptional regulation of gene expression. They have been shown to be involved not only in cell differentiation and proliferation but also to serve as key regulators of metabolism, controlling essential metabolic pathways that support energy balance and cellular function [26]. Metabolism encompasses the biochemical processes essential for proper cellular function. Several studies indicate that miRNAs coordinately regulate pathways involved in glycolysis, lipid metabolism, cholesterol biosynthesis, and insulin sensitivity, among others [26].

Regarding lipid metabolism and adipogenesis, miRNAs are recognized as key regulators of metabolism during exercise and in response to dietary changes, particularly in skeletal muscle, where they regulate genes involved in muscle contraction and energy metabolism [27]. miR-143 is known to promote adipogenesis. This miRNA regulates differentiation by influencing the MAPK signaling pathway, thereby facilitating the proliferation and differentiation of preadipocytes into mature adipocytes, while also contributing to glucose homeostasis and increased lipid accumulation [28,29]. In contrast, miR-155 has been shown to negatively regulate adipogenesis by binding to the mRNA of C/EBPβ factor [30]. Alterations in the expression of these miRNAs can affect muscle adaptation. Furthermore, it is also well known that miRNAs do not regulate the adipogenesis process solely through direct pathways. For instance, miR-27 can target the prohibitin protein (PHB)—which is closely associated with strongly related to mitochondrial biogenesis—and consequently downregulate PPARγ expression as a consequence, leading to mitochondrial dysfunction and reduced lipid metabolism in 3T3-L1 cells [31] and lipid metabolism reduction. Another example of this mechanism occurs through inflammatory pathways. When mature adipocytes become hypertrophic, they secrete chemokine (C-C motif) ligand 2 (CCL2) to recruit M1 macrophages. In this context, miRNAs such as miR-126 and miR-193b can directly target CCL2 mRNA or indirectly modulate its levels through transcription factor circuits [32] to attenuate this proinflammatory environment.

The importance of miRNAs in metabolic regulation is also evident in pathological conditions, where low-grade chronic inflammation alters their expression profile as the system attempts to maintain metabolic homeostasis. Variations in miR-221/222 and miR-155, for example, may participate in the crosstalk between obesity-related inflammation, insulin resistance, and other obesity-associated morbidities [33], reflecting the complexity of these small molecules and their critical role in the adipogenesis process.

In addition, Aloe-emodin has shown glucose-lowering efficacy by activating PI3K and GLUT4 proteins, which are essential in the adipogenesis process, while maintaining the capacity to modulate metabolic markers such as PPARγ without inducing full cell differentiation [34]. These findings suggest that Aloe-emodin may upregulate gene expression to compensate for altered glucose uptake, while simultaneously modulating miRNA expression that interacts, which cross-talks with these pathways. Consequently, this study aimed to explore the miRNA expression profile in the presence of Aloe-emodin alongside key adipogenic markers. Taken together, the disruption of the expression of these small regulators can alter multiple metabolic pathways, potentially exacerbating clinical conditions.

## 2. Materials and Methods

### 2.1. Bibliometric Search

The bibliometric analysis was conducted on 15 February 2026, using the PubMed database. The search strategy applied was: ((“MicroRNAs” [MeSH]) AND (“Adipogenesis” [MeSH]). Only articles published in the last 10 years (2015–2025) were included, with no language restrictions. The complete set of results was exported as a PubMed format (.txt) file, containing all metadata (title, abstract, keywords, MeSH terms). The file was imported into VOSviewer software (version 1.6.20, Leiden University, The Netherlands). Co-occurrence analysis of MeSH terms and keywords was performed using the full counting method, with a minimum threshold of 5 occurrences per term. The association strength method was used for normalization. The resulting term co-occurrence map was visualized and analyzed.

### 2.2. In Vitro Model

The 3T3-L1 cell line (ATCC^®^ CL-173™, Manassas, VA, USA) was used. Cell proliferation and differentiation were carried out following the methodology described by Constant et al. [35] using HyClone™ RPMI 1640 medium: Liquid (Cytiva, SH30027.01, Logan, UT, USA) supplemented with 10% Fetal Bovine Serum (BioWest—Fetal Bovine Serum (USA Origin) S1520, Nuaillé, France) in T25 flasks. The T25 flask medium was changed every three days, and cells were maintained under the following conditions: 37 °C, 95% air, 5% CO_2_, and 2.0 g/L of Glucose.

### 2.3. Cellular Differentiation and Aloe-Emodin Treatment

Upon reaching approximately 80% confluency, the cell biomass was harvested in a 15 mL conical tube by centrifugation at 2500 rpm for 10 min. The harvested cells were then seeded in a six-well plate at a density of 30,000 cells/well. Cell differentiation was performed using the commercial Adipogenesis Assay Kit (Cayman Chemical, 10006908, Ann Arbor, MI, USA) according to the manufacturer’s instructions. On day 3, the medium was changed by adding 4 µLA total of IBMX 1000X, 4 µL of insulin solution 1000X, and 4 µL of dexamethasone solution 1000X per 2 mL of medium per well. On day 7, only 4 µL of insulin solution 1000X was added per 2 mL of medium per well. Four groups were analyzed across three independent experiments (in a six-well plate in triplicate): control group (fully differentiated cells without Aloe-emodin treatment), added from day 0 and at each medium change); (c) Aloe-emodin group with differentiation medium (40 µM, dissolved in DMSO (0.23% *v*/*v*); (c) Aloe-emodin group without differentiation medium (40 µM, dissolved in DMSO (0.23% *v*/*v*), added from day 0 and at each medium change; (d) TNF-α group with differentiation medium (5 ng/mL, added from day 0 and at each medium change), as previously described [36]. All groups were maintained and evaluated up to day 10 of treatment, which resulted in robust differentiation. For the Aloe-emodin vera-Emodin treatment, we adapted the methodology described by Subash-Babu et al. [7] by doubling the final concentration used for differentiation. This dose ensures safe cell viability, avoiding potential cytotoxic effects while maintaining the targeted differentiation response, in accordance with the reported values for differentiation, keeping it within the IC50 values: 24 h—418 ± 13.2 µM; 48 h—390 ± 10.5 µM range.

### 2.4. Oil Red Staining

Staining was performed using the commercial Adipogenesis Assay Kit (Cayman Chemical, 10006908, Ann Arbor, MI, USA) according to the manufacturer’s instructions, with the following modifications to the washing steps: the “Wash solution” was replaced with 1 mL of 1X PBS (Gold Biotechnology^®^, St. Louis, MO, USA). Finally, 200 μL of the suspension was transferred in quintuplicate into 5 replicates in a 96-well plate for each independent experiment, and absorbance was measured at 492 nm.

### 2.5. RNA Extraction

Total RNA extraction was performed for each independent experiment directly from cells using TRIzol^®^ reagent (Carlsbad, CA, USA), followed by chloroform addition according to the manufacturer’s instructions. The mixture was homogenized and incubated at room temperature for 5 min, then centrifuged at 13,000 rpm for 15 min at 4 °C. The clear aqueous upper phase was collected, and 500 µL of isopropanol was added. The mixture was incubated at −20 °C for 15 min to precipitate the RNA, which was then pelleted by centrifugation at 13,000 rpm for 15 min at 4 °C. The pellet was washed with 75% ethanol and centrifuged at 13,000 rpm for 5 min at 4 °C. After drying, the RNA pellet was resuspended in nuclease-free water and stored at −80 °C until further analyses, with aliquots maintained at −20 °C as needed. purity was assessed using a NanoDrop spectrophotometer (NanoDrop Technologies, Waltham, MA, USA). Preliminary RNA integrity was evaluated by agarose gel electrophoresis (2% agarose/TAE), with bands visualized on a UV transilluminator, and 1 mL of RNase inhibitor (RNasin^®^, Promega, Madison, WI, USA) was added. Final RNA integrity was validated by determining the RNA integrity number (RIN) using an Agilent 2100 Bioanalyzer (Agilent, Santa Clara, CA, USA)with the RNA Nano chip.

### 2.6. RNA-Sequencing

Messenger RNA (mRNA) enrichment was performed using the NEBNext Poly (A) mRNA Magnetic Isolation Module across three independent experiments. Library construction was carried out using the MGIEasy RNA library prep kit (MGI Tech Co., Ltd., Shenzhen, China) to generate inserts of approximately 350 bp, which were then labeled with the MGIEasy DNA Adapters-96 (plate) kit (MGI Tech Co., Ltd., Shenzhen, China). Quality control of the constructed libraries was verified on an Agilent 2100 Bioanalyzer system using the DNA 1000 chip. High-throughput sequencing was conducted at the Genomic Services Laboratory (LabSerGen) of the Advanced Genomics Unit at Cinvestav, using the MGISeq 2000 platform (MGI Tech Co., Ltd., Shenzhen, China). Sequencing was performed in paired-end (PE) 2 × 150 bp mode with a small flow cell (FCS).

### 2.7. Bioinformatics Data Processing and Quantification

All analyses were performed on the Linux-based ayotochtli server running Debian GNU/Linux 12 (Bookworm), equipped with two Intel Xeon Gold 6136 processors (24 physical cores and 48 logical threads) and 251 GB of RAM. The system also featured an NVIDIA Tesla T4 GPU with 15 GB of VRAM (CUDA 12.4). Primary analysis of the raw data was conducted using the nf-core/rnaseq pipeline, executed via the Nextflow workflow manager [37] and standardized under the nf-core community framework [38], utilizing Docker containers to ensure reproducibility.

Quality control of the raw reads was initially assessed with FastQC [39]. For data cleaning and filtering—specifically the removal of low-quality sequences and adapter contamination—SOAPnuke tool (BGI, Shenzhen, China) [40] was used. The removal of the following adapters, corresponding to MGI chemistry, was explicitly configured:

Forward filterfilter: AAGTCGGAGGCCAAGCGGTCTTAGGAAGACAA

Reverse filterfilter: AAGTCGGATCGTAGCCATGTCGTTCTGTGAGCCAAGGAGTTG

Processed and clean reads were aligned against the complete mouse reference genome (Mus musculus, assembly GRCm39). Alignment was performed using STAR (version 2.7.11b) [41], supplemented by Salmon [42] for rapid and robust quantification against transcript- and gene-level biases. Merged gene-level count matrices were generated for subsequent analyses.

### 2.8. Differential Analysis

Differential expression analysis was performed using the nf-core/differential abundance pipeline [37], using the Salmon-generated count matrix as input. Count normalization and differential analysis were carried out using the DESeq2 package [43] within the R environment. Experimental group definitions included Control, Treatment, and Undifferentiated conditions, and pairwise contrasts were performed as specified in the experimental design. Genes with an adjusted *p*-value (FDR) < 0.05 and an absolute Log_2_FC > 0 were considered differentially expressed.

### 2.9. Functional Enrichment Analysis (GSEAGSEA)

Gene Set Enrichment Analysis (GSEA) was performed [44] to identify altered biological pathways and cellular processes, utilizing the Hallmark gene set collection from the Molecular Signatures Database (MSigDB) adapted from the murine model [44]. The analysis employed the log2_Ratio_of_Classes metric to rank genes and assess the overrepresentation of key biological pathways associated with the studied phenotypes.

### 2.10. cDNA Synthesis and qPCR

Reverse transcription (RT) was performed using M-MLV Reverse Transcriptase (Invitrogen, Carlsbad, CA, USA; 28025021) according to the manufacturer’s instructions, using samples from three independent experiments. The cDNA was stored at −25 °C. qPCR was carried out using 2x Maxima SYBR Green no ROX (Thermo Scientific, Baltics, Vilnius, Lithuania). The following genes were evaluated: Rplp0, used as the reference gene for data normalization by the 2^−∆∆Ct^ method; *Pparg1*, *Cebpb*, and *Fabp4*, as adipogenesis markers; *Adipoq* and *Scl2a4*, as adipocyte markers. The primers used for this study are listed in Table 1. Samples with undetermined Ct values were treated as “not detected” for qualitative purposes, and these were not assigned quantitative Fold Change values.

### 2.11. miRNA Analysis

cDNA synthesis for miRNA detection was performed using the Custom TaqMan™ Small RNA Assay Kit (Applied Biosystems, Pleasanton, CA, USA) for miR-143 (Assay ID: 463509 mmu-miR-143*, Thermo Fisher Scientific, Waltham, MA, USA) and miR-155 (Assay ID: 002571 mmu-miR-155-5p, Thermo Fisher Scientific, Waltham, MA, USA), according to the manufacturer’s instructions, using samples from three independent experiments. U6 snRNA (Assay ID: 001973 U6 snRNA, Thermo Fisher Scientific, Waltham, MA, USA) was used as the endogenous control for data normalization by the 2^−∆∆Ct^ method. qPCR was carried out using the QuantiNova™ Probe PCR Kit (QIAGEN, Hilden, Germany) according to the manufacturer’s instructions, with the specific probes provided in the Custom TaqMan™ Small RNA Assay Kit (Applied Biosystems, Pleasanton, CA, USA). Detection was performed on a CFX Opus 96 Real-Time PCR System (Bio-Rad, Cat. Number 2011319, Hercules, CA, USA) and the analysis was conducted using CFX Maestro Software. The sequence of these miRNAs and snRNA are listed in Table 2.

### 2.12. Statistical Analysis

Statistical analyses was performed using R software (version 4.6.0 (24 April 2026)) with the packages “tidyverse,” “rstatix,” “ggpubr,” and “openxlsx,”. All data are expressed as mean ± standard error of the mean (SEM). Normality of the data was assessed using the Shapiro–Wilk test (*n* < 50). For comparisons involving more than three groups with normally distributed data, a one-way ANOVA was used, followed by a Tukey–Kramer post hoc test. For non-normally distributed data, the Kruskal–Wallis test was performed with Dunn post hoc test. Differences were considered statistically significant at *p* < 0.05 and highly significant at *p* < 0.005. Complementary to this, a Log_2_Log Fold Change (LFC) analysis was performed to visualize the expression profiles of miRNAs and transcription factors following up on treatment with Aloe-emodin vera-Emodin and TNF-α.

## 3. Results

### 3.1. Bibliometric Analysis

**Figure 1:**
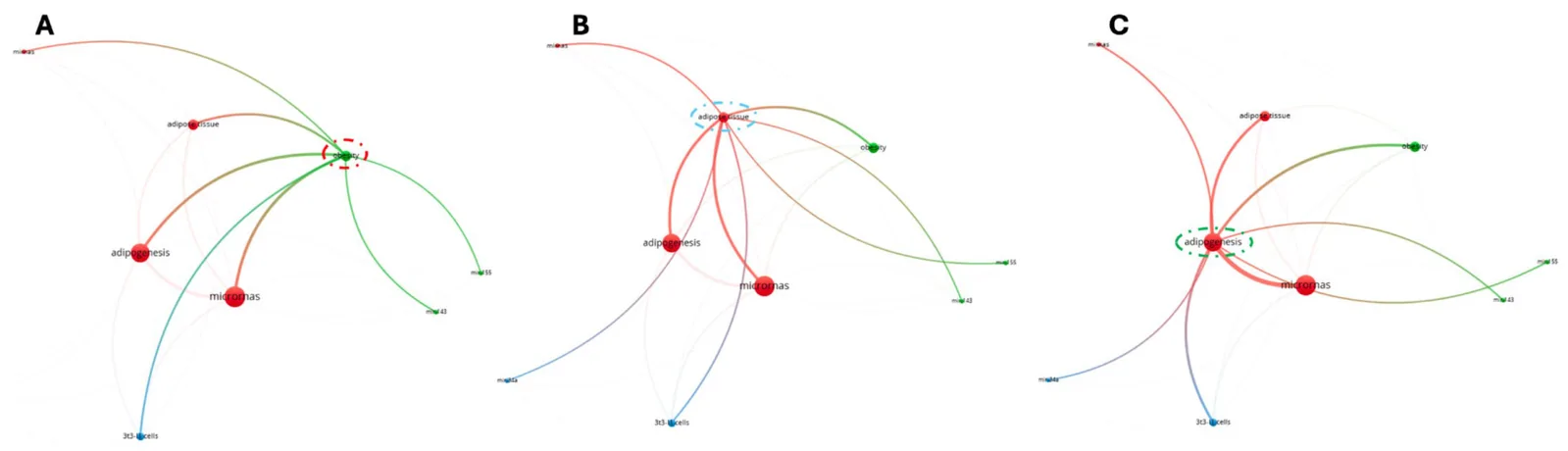
Terms used as convergence points to determine the relationship between words and existing literature. (**A**) “obesity” (red) as the first convergence point. (**B**) “adipose tissue” (blue) as the second convergence point. (**C**) “adipogenesis” (green) as the third convergence point. Each term displays different branches where relationships exist among the selected terms, with each branch serving as a “path” to identify common terminology and the point of convergence.

For the 10-year period (2015–2025), a PubMed search using the MeSH terms “MicroRNAs” and “Adipogenesis” yielded a total of 755 articles. The search output file (.txt) was uploaded to VOSviewer software, and a minimum occurrence threshold of five times was set for the following terms: microRNAs, adipogenesis, obesity, adipose tissue, and 3T3-L1, as well as other miRNAs identified by the software based on the bibliography. A total of 277 terms were displayed, from which each of the previously mentioned terms was manually selected. Once the analysis was complete, the software generated a map showing the associations among the terms with the highest occurrence in the literature. The selection of terms showing association on the map was performed by choosing the following complementary terms: “obesity” (Figure 1A), which branched into miR-155 and miR-143; “adipose tissue” (Figure 1B), which branched into miR-155, miR-143, and miR-34a; and finally, “adipogenesis” (Figure 1C), which branched into miR-155, miR-143, and miR-34a, as these terms exhibit the highest number of associations and help identify convergence points on the map:

Once we confirmed that the previously described miRNAs were common to our three convergence terms, each of them was evaluated against all other displayed terms to ensure full co-occurrence. As a result, only miR-143 and miR-155 met this condition, making them the selected candidates for further analysis.

### 3.2. RNA-Seq and Aloe-Emodin Effect

Messenger RNA (mRNA) enrichment was performed using the NEBNext Poly (A) mRNA Magnetic Isolation Module, ensuring selective capture of polyadenylated transcripts while minimizing ribosomal RNA contamination, as the study aimed to detect differential gene expressions and low-abundance regulatory RNAs. Library construction was carried out using the MGIEasy RNA library preparation kit (MGI Tech Co., Ltd., Shenzhen, China), generating insert sizes of approximately 350 bp, a configuration optimized for paired-end sequencing and reliable transcript reconstruction. The sequencing results revealed the presence of the long non-coding RNA Mir-155hg, exhibiting a Log_2_ Fold Change = 2.56 (FC = 5.89, FDR = 0.003) (Supplementary Materials). The identification of Mir-155hg confirms the sensitivity and resolution of the RNA-Seq workflow, as this lncRNA is known to function as a transcriptional precursor and regulatory hub associated with inflammatory signaling and metabolic dysregulation.

### 3.3. Aloe-Emodin Effect in Cell Differentiation and Oil Red O Staining at 10 Days

**Figure 2:**
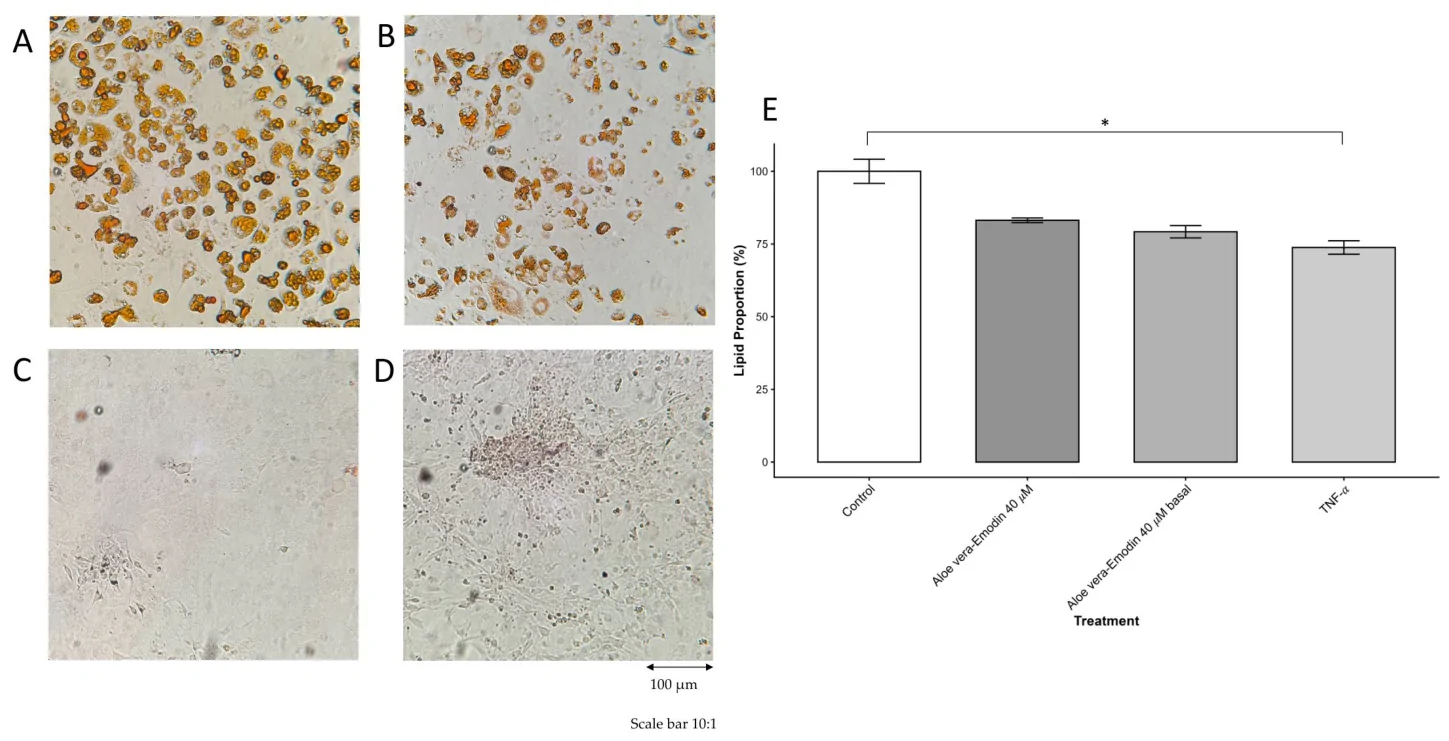
Cell differentiation and Oil Red O staining. (**A**) Cell morphology of cell differentiation with Oil Red O in the differentiated control group; it can be observed that there is a higher density of mature adipocytes. (**B**) Aloe-emodin at 40 µM with differentiation medium: a higher density of precursor cells and a lower number of mature adipocytes are observed. (**C**) Group treated only with Aloe-emodin at 40 µM: no morphological changes are observed in the 3T3-L1 cell line. (**D**) Group treated with TNF-α as a negative control; few changes in cell morphology are observed, indicating that the differentiation process was halted. (**E**) Statistical analysis of the intracellular lipid stain assay in the different groups; TNF-α was the only one with significant differences (*p* < 0.05). Statistical test: Kruskal–Wallis with Dunn post hoc test. * *p* < 0.05. Images were captured using a 10× objective lens (NA: 0.25) on a light microscope. Data are representative of at least 5 randomly selected independent fields per well from three independent experiments.

Treatment with 40 µM Aloe-emodin and differentiation medium (Figure 2B) showed notable differences in the number of mature adipocytes compared to the differentiated control group (Figure 2A); however, no significant differences were observed between these two groups (Figure 2E). For the group treated with Aloe-emodin in basal medium (Figure 2C), no significant morphological changes were observed relative to the differentiated control group (Figure 2A,E). Finally, the group treated with TNF-α and differentiation medium (Figure 2D) showed significant differences (*p* < 0.05) compared to the control group, with a cluster of cells exhibiting reduced size and lower dye uptake.

### 3.4. qPCR Assay and Gene Expression

**Figure 3:**
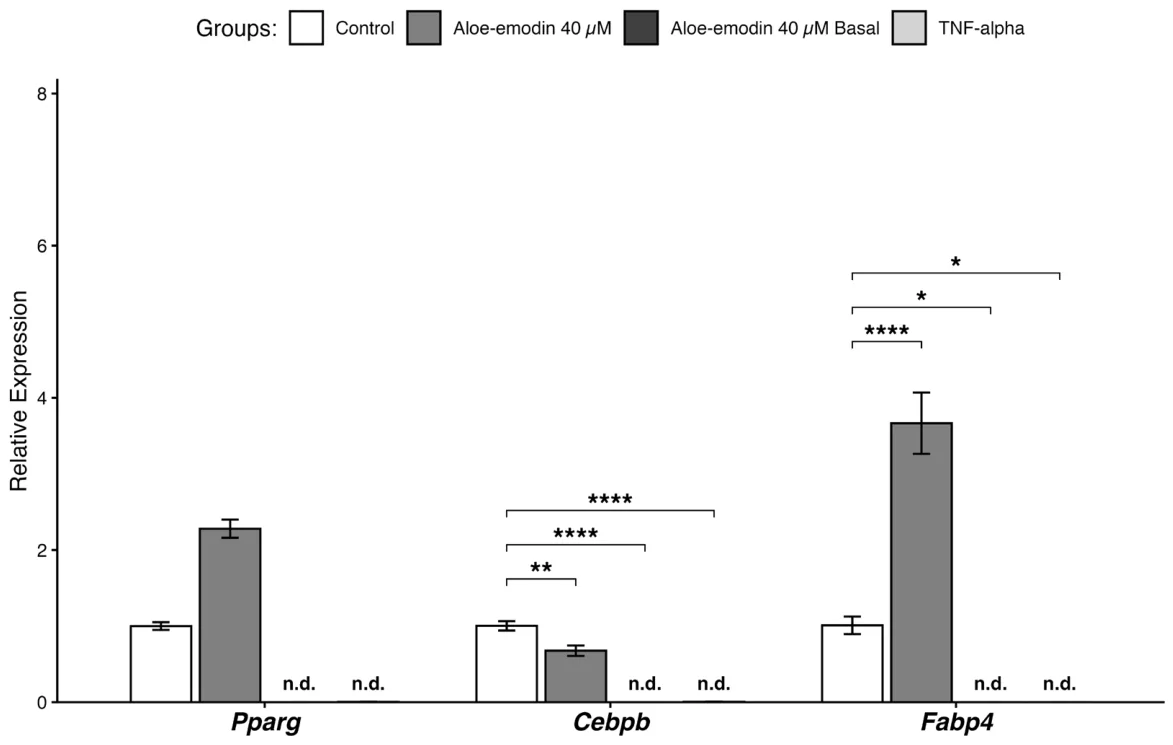
Expression of adipogenesis genes at 10 days of differentiation. Gene expression is absent in the group treated with Aloe-emodin (without differentiation medium) and TNF-α. On the other hand, Pparg and Fabp4 are upregulated in the Aloe-emodin group with differentiation medium, whereas Cebpb is downregulated relative to the differentiated control group. Results are shown as three independent experiments (*n* = 3 replicates per group). Statistical analysis: One-way ANOVA with Tukey–Kramer post hoc test. * *p* < 0.05, ** *p* < 0.005, **** *p* < 0.0005, “n.d.”: not determined.

Adipogenesis-related gene expression was measured 10 days after the initiation of differentiation. Cebpb showed highly significant differences (*p* < 0.005) in the differentiated control group, and very highly significant differences (*p* < 0.0005) in the group treated with 40 µM Aloe-emodin and basal medium, as well as the TNF-α with differentiation medium group, indicating failed adipogenic commitment (Figure 3). No significant differences were found for the group treated with 40 µM Aloe-emodin in differentiation medium. Pparg showed no significant differences in any of the groups; however, expression of this gene was not detected in the 40 µM Aloe-emodin with basal medium group, nor in the TNF-α and differentiation medium group (Figure 3).

Finally, Fbp4Fbp4 showed significant differences (*p* < 0.05) in the 40 µM Aloe-emodin with basal medium group, as well as the TNF-α with differentiation medium group, because—as with the previous genes—its expression was not detected (Figure 3). The differentiated control group showed very highly significant differences (*p* < 0.0005), while the 40 µM Aloe-emodin with differentiation medium group showed no significant differences (Figure 3).

**Figure 4:**
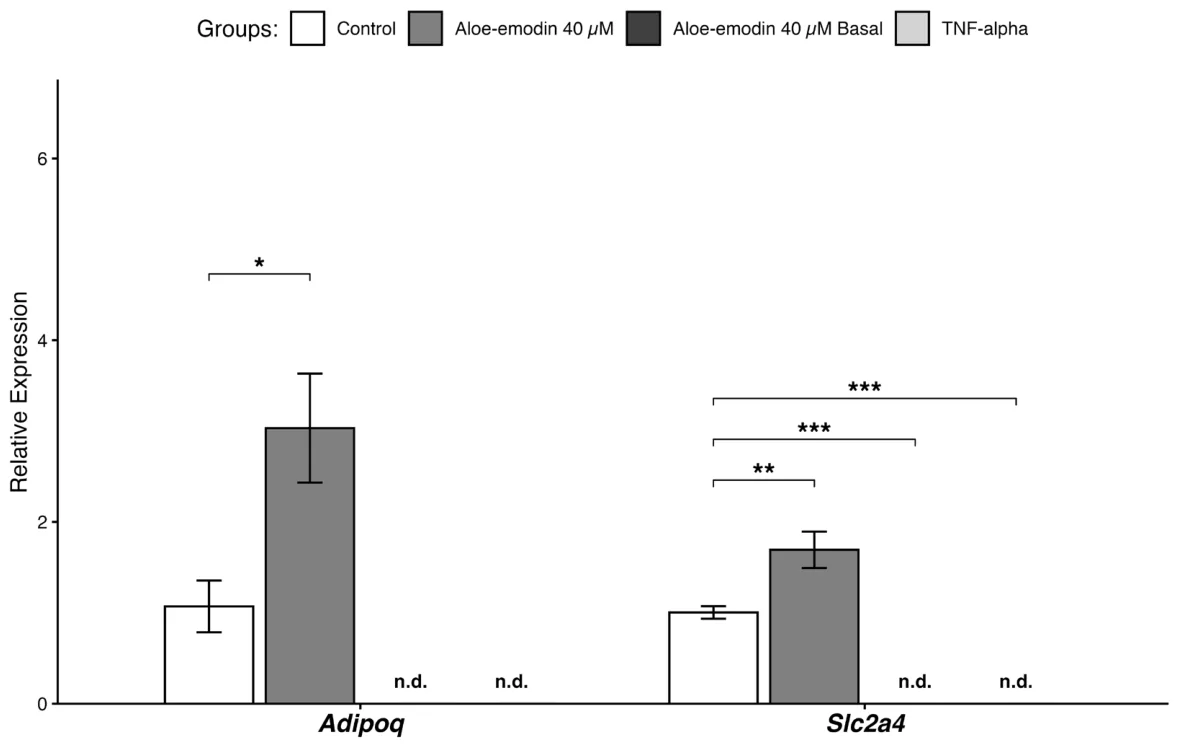
Expression of *Adipoq* and *Slc2a4* genes at 10 days of differentiation. It was observed that gene expression was not detected in the 40 µM Aloe-emodin vera-Emodin + basal medium and TNF-α groups. The group treated with 40 µM Aloe-emodin vera-Emodin and differentiation medium showed overexpression of both genes. Results are shown as three independent experiments (*n* = 3 replicates per group). Statistical analysis: One-way ANOVA with Tukey–Kramer post hoc test. * *p* < 0.05, ** *p* < 0.005, *** *p* < 0.0005, “n.d.”: not determined.

The expression of the *Adipoq* and *Scl2a4* genes, which served as markers for mature adipocytes, was measured 10 days after the initiation of differentiation. The group treated with 40 µM Aloe-emodin in vera-Emodin and differentiation medium showed no significant differences in *Adipoq* or *Scl2a4* expression compared to the control group (Figure 4). In contrast, the group treated with 40 µM Aloe-emodin in vera-Emodin and basal medium showed significant differences (*p* < 0.05) for Adipoq and highly significant differences (*p* < 0.005) for Scl2a4; however, this resulted from the fact that no gene expression was detected in these samples (Figure 4). A similar trend was observed for the group treated with TNF-α in a differentiation medium, which only showed highly significant differences for Scl2a4 (*p* < 0.005), while neither Scl2a4 nor Adipoq expression was detected at day 10 (Figure 4).

### 3.5. miRNA Expression

**Figure 5:**
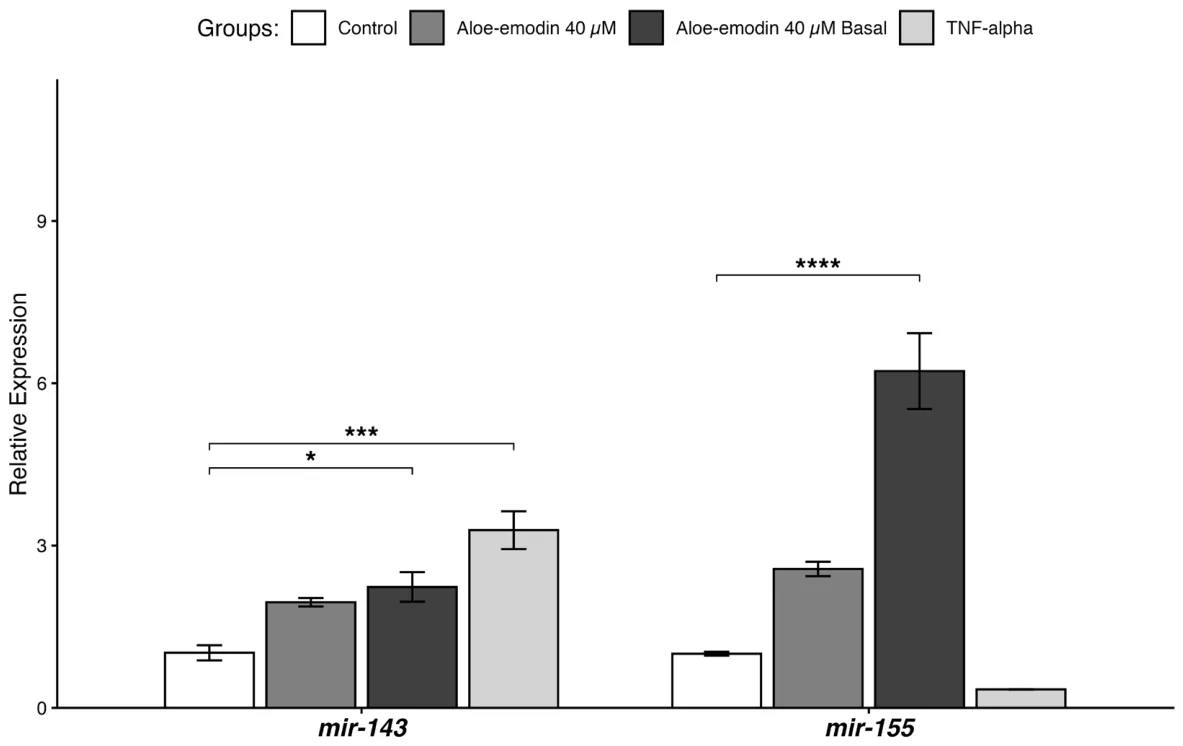
Expression of miR-143 and miR-155 at 10 days of differentiation. It was observed that miR-143 is overexpressed in all groups, while miR-155 only shows overexpression in the 40 µM Aloe-emodin differentiation medium and basal medium groups. Data represent three independent experiments for each miRNA expression analysis (*n* = 3 per group). Statistical analysis: One-way ANOVA with Tukey–Kramer post hoc test. * *p* < 0.05, *** *p* < 0.005, **** *p* < 0.0005.

As observed in Figure 5, the expression of miR-143 and miR-155 was analyzed at 10 days of differentiation. miR-143 showed significant (*p* < 0.05) and highly significant (*p* < 0.005) differences in the 40 µM Aloe-emodin with basal medium and TNF-α groups, respectively, compared to the control group. Regarding miR-155, highly significant differences (*p* < 0.005) were observed in the 40 µM Aloe-emodin with basal medium group compared to the control. The remaining groups showed no significant differences.

**Figure 6:**
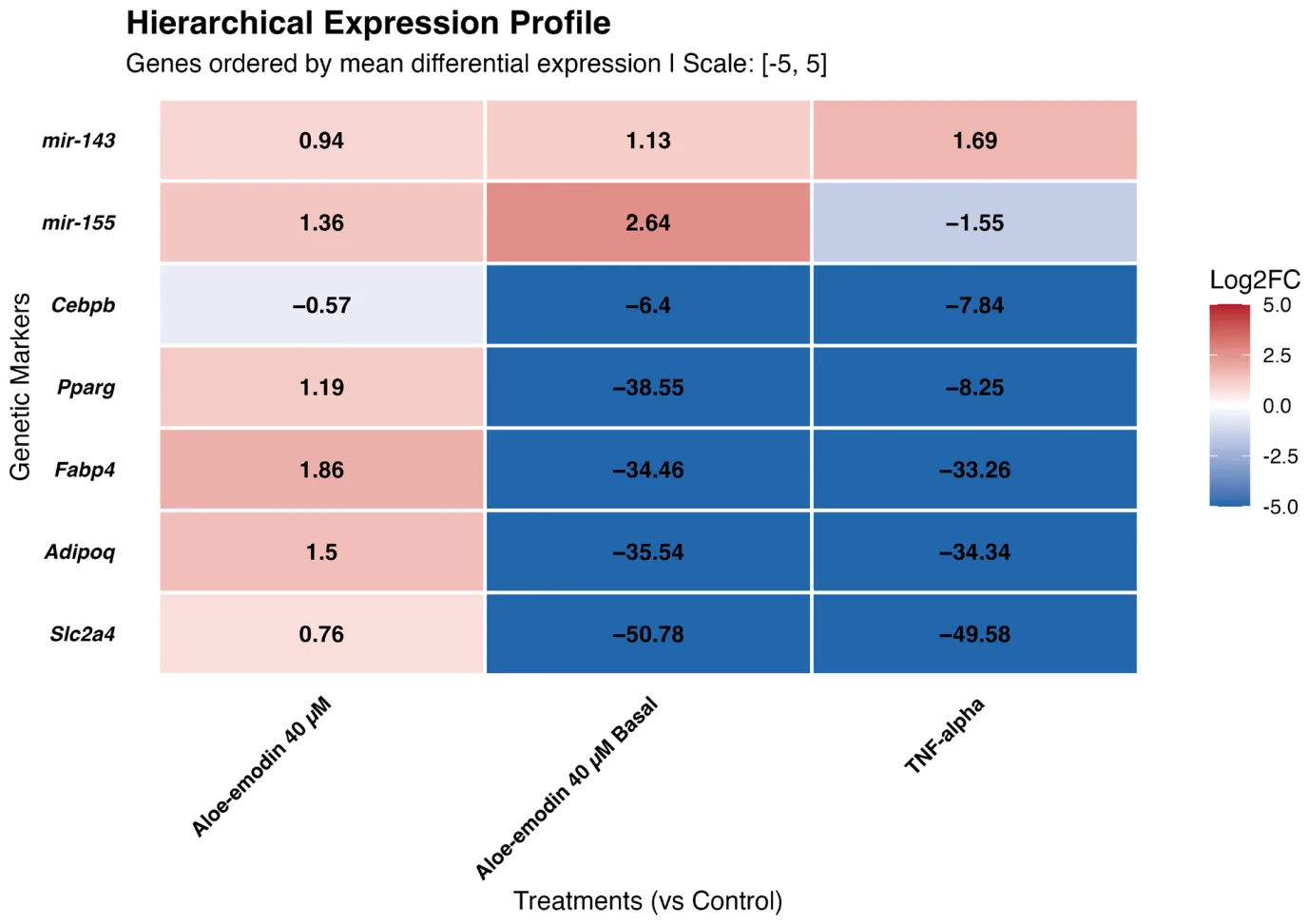
Heat map of the gene expression profile. Red indicates upregulated expression, while blue indicates downregulated/no expression. Each box represents the Fold Change in gene expression in comparison with the control group. It is notable that Aloe-emodin vera-Emodin (without differentiation media) and TNF-α have no adipogenic properties in the 3T3-L1 cell line. The expression was not detected and should be interpreted qualitatively, not as experimentally measured fold changes.

The Log_2_Log Fold Change analysis assay illustrates gene expression changes in response to treatment with Aloe-emodin and TNF-α treatment effect (Figure 6) at 10 days of differentiation (Figure 6). Adipogenesis and adipocyte markers remain upregulated exclusively under treatment with Aloe-emodin in the presence of Aloe-emodin with differentiation medium (except for Cebpb). Notably, both miR-143 and miR-155 were upregulated by the two Aloe-emodin treatments (with and without differentiation medium). Conversely, following TNF-α treatment, all adipogenic and adipocyte markers were not detected; under these conditions, miR-155 was downregulated, whereas miR-143 was upregulated.

## 4. Discussion

The implementation of high-throughput RNA sequencing (RNA-Seq) was strategically selected as a complementary approach to provide a global and unbiased overview of transcriptional alterations associated with the studied condition. Given that the experimental design aimed to identify molecular pathways and regulatory elements potentially involved in the observed biological effects, RNA-Seq represents a robust and sensitive method for detecting both coding and non-coding RNA species. Notably, the detection of Mir-155hg overexpression provides mechanistic support for the biological observations obtained through complementary assays, as this lncRNA is processed to yield miR-155. This finding positions Mir-155hg as a potential molecular mediator within the studied model, underscoring the relevance of this approach and supporting its role as an essential component in the integrative interpretation of the experimental data.

The present results indicate that treatment with 40 µM of Aloe-emodin in the presence of differentiation medium produced observable morphological changes in mature adipocytes compared to the differentiated control group; however, these alterations did not reach statistical significance. This suggests that, under the conditions evaluated, 40 µM of Aloe-emodin does not exert a strong inhibitory or stimulatory effect on adipocyte differentiation when administered alongside a complete adipogenic cocktail. Previous studies using the 3T3-L1 adipocyte model have reported that emodin and related anthraquinone compounds can exert dose-dependent inhibitory effects on adipogenesis, primarily through the suppression of lipid accumulation and adipogenic gene expression. For example, Zhang et al. [45] demonstrated that Aloe-emodin inhibited adipocyte differentiation and fatty acid synthase activity in 3T3-L1 cells at low micromolar concentrations, highlighting a potential anti-adipogenic role for this compound. Similarly, Aloe-emodin derivatives have been shown to suppress adipogenesis via modulation of insulin-dependent signaling pathways and inhibition of lipid accumulation [34,45]. Likewise, the presence of significant differences in four of the evaluated adipogenic genes (Cebpb, Fabp4, Adipoq, and Slc2a4) upon treatment with Aloe-emodin combined with differentiation medium indicates a strong relationship between the treatment and cellular differentiation. Previous reports indicate that emodin selectively inhibits adipogenesis induced by specific metabolic signals rather than acting as a universal differentiation blocker [46]. This aligns with the overexpression of Pparg and Fabp4, which are essential for terminal differentiation; meanwhile, the fact that Cebpb remains below the differentiated control group reinforces its role as an early recruiter of Pparg. Notably, treatment with Aloe-emodin under basal medium conditions did not induce morphological or quantitative changes in adipocyte number or lipid staining relative to controls, reinforcing the possibility that Aloe-emodin alone may be insufficient to trigger adipogenic commitment in preadipocytes. This observation is consistent with in vitro data suggesting that *Aloe vera* extracts and emodin primarily modulate adipocyte metabolism and the anti-inflammatory state, rather than initiating differentiation per se [47]. Conversely, exposure to TNF-α in the presence of differentiation medium resulted in significant reductions in adipocyte size and dye uptake, accompanied by clear morphological alterations. These findings are consistent with the extensive literature demonstrating that TNF-α is a potent negative regulator of adipogenesis in 3T3 L1 cells. TNF-α suppresses adipocyte differentiation through repression of PPARγ and C/EBPα expression and activation of anti-adipogenic pathways such as Wnt/β-catenin and NF κB signaling. The reduced lipid accumulation observed in our study is therefore indicative of impaired adipogenic maturation rather than cytotoxicity [48]. Overall, these results suggest that Aloe-emodin does not significantly interfere with adipocyte differentiation under strong adipogenic stimulation, whereas TNF-α effectively disrupts adipocyte maturation. The data support a model in which emodin may exert context-dependent metabolic or anti-inflammatory effects rather than acting as a direct inhibitor of adipogenesis. Further studies evaluating gene expression profiles, lipid metabolic flux, and inflammatory signaling pathways may help clarify the precise role of emodin in adipocyte biology, particularly under conditions of metabolic stress rather than maximal differentiation.

The expression of Adipoq and Slc2a4Slc2a4, two well-established markers of mature adipocytes, was evaluated 10 days after the initiation of differentiation. The significant differences in Adipoq and Slc2a4Slc2a4 expression in the group treated with 40 µM Aloe-emodin in differentiation medium relative to the control suggest that, under strong adipogenic stimuli, emodin does affect mature adipocytes. This finding is consistent with reports indicating that adipogenic differentiation driven by a full hormonal cocktail can override moderate modulatory effects of phytochemicals on downstream adipocyte markers [45,47]. In contrast, the lack of detectable Adipoq and Slc2a4 expression in the group treated with Aloe-emodin under basal conditions and in the TNF-α-treated group under differentiation conditions indicates a failure to achieve terminal adipocyte differentiation. The significant and highly significant differences observed in these groups do not reflect upregulation or downregulation per se, but rather the absence of adipocyte-specific gene expression, which strongly suggests impaired adipogenic commitment. These findings are in line with previous studies demonstrating that Adipoq and Slc2a4Slc2a4 expression are strictly dependent on the activation of the canonical adipogenic transcriptional cascade involving Cebpb, Cebpba, and Pparg [49,50]. This suggests that while Cebpb has already fulfilled its initiating function, Pparg fully consolidates as the master factor driving terminal differentiation at this point in the assay—a profile consistently supported by the behavior of downstream terminal markers such as Adipoq and Slc2a4—despite the differences in the proportion of mature adipocytes relative to the differentiated control group. The suppressive effect observed in the TNF-α treated group is highly consistent with the extensive literature identifying TNF-α as a potent anti-adipogenic cytokine. TNF-α has been shown to inhibit adipogenesis by preventing the induction of C/EBPβ and PPAR γ, activating NF-κB signaling, and stabilizing β-catenin, ultimately blocking the transcriptional program required for adipocyte maturation [48,51]. The absence of Adipoq, Slc2a4, Fabp4, and Pparg expression in this group therefore supports the reliability of the experimental model and confirms effective inhibition of adipocyte differentiation. Regarding early and intermediate adipogenic markers, Cebpb expression showed highly significant differences in the control group, consistent with its transient but essential role during the early phase of adipogenesis. Notably, the absence of detectable Cebpb expression in the Aloe-emodin with basal medium group and the TNF-α-treated group suggests that both conditions interfere at an early stage of adipogenic programming. This observation aligns with studies demonstrating that blocking Cebpb expression effectively prevents downstream activation of Pparg and Fabp4Fabp4, resulting in failure of adipocyte maturation [52]. The absence of Pparg expression in the Aloe-emodin basal medium group contrasts with reports describing emodin as an inhibitor of adipogenesis through modulation of Pparg activity rather than complete suppression of its expression [46,53]. This discrepancy may be explained by differences in experimental context, including the absence of adipogenic inducers and the timing of gene expression analysis. Under basal conditions, emodin may act as a metabolic modulator rather than an active anti-adipogenic agent, indirectly suppressing adipogenic markers by preventing commitment rather than repressing transcriptional activity. Similarly, the lack of Fabp4Fabp4 expression in the Aloe-emodin basal group and the TNF-α-treated group further supports the conclusion that these conditions do not possess the properties required to induce adipogenesis. FABP4 is a late adipogenic marker tightly linked to lipid accumulation and adipocyte functionality, and its absence is typically associated with incomplete or aborted adipogenesis [54]. Taken together, these findings indicate that Aloe-emodin does not significantly alter adipogenic gene expression when administered in the presence of a full differentiation cocktail but may impair adipogenic commitment under basal conditions. In contrast, TNF-α robustly suppresses the adipogenic program, validating its role as a positive control for anti-adipogenic signaling. These results suggest that emodin may exert context-dependent effects, potentially acting as a metabolic or anti-inflammatory modulator rather than a direct inhibitor of adipocyte differentiation, a notion supported by studies linking emodin to AMPK activation, insulin signaling modulation, and inflammatory pathway regulation [34,53].

The analysis of miR-143 and miR-155 expression at day 10 of differentiation provides relevant insight into the microRNA-mediated regulation of adipogenesis under inflammatory and phytochemical modulation. miR-143 is widely recognized as a pro-adipogenic microRNA, whose expression increases during adipocyte differentiation and contributes to lipid accumulation and adipocyte maturation. In this context, the observation that miR-143 showed significant and highly significant differences in the 40 µM Aloe-emodin with differentiation medium group and the TNF-α group, respectively, compared to the control, suggests that both treatments interfere with adipogenic regulatory mechanisms at the post-transcriptional level. Previous studies have demonstrated that miR-143 promotes adipocyte differentiation by modulating ERK5 signaling and facilitating PPAR-γ activation, and that its downregulation impairs adipogenesis [28]. Therefore, the strong alteration of miR-143 expression observed in the TNF-α-treated group is consistent with the well-established anti-adipogenic role of TNF-α, which suppresses adipocyte differentiation through activation of inflammatory pathways such as NF-κB and Wnt/β-catenin, indirectly affecting adipogenesis-associated microRNAs [48,51]. The significant modulation of miR-143 in the Aloe-emodin with differentiation medium group, despite the absence of marked suppression of terminal adipogenic markers in previous results, suggests that emodin may modulate microRNA expression without completely inhibiting adipocyte maturation. This observation aligns with reports indicating that emodin and related anthraquinone compounds exert context-dependent metabolic effects, influencing regulatory signaling rather than uniformly blocking adipogenesis [45,46]. In contrast, miR-155 expression was significantly increased only in the group treated with 40 µM Aloe-emodin under basal conditions, whereas no significant differences were detected in the remaining groups. miR-155 is a well-characterized pro-inflammatory and anti-adipogenic microRNA, known to impair adipocyte differentiation and insulin signaling, particularly in inflamed adipose tissue [30,55]. The selective induction of miR-155 under basal conditions suggests that emodin may promote an inflammatory-associated microRNA profile when adipogenic cues are absent, potentially preventing early adipogenic commitment. Interestingly, the absence of significant miR-155 modulation in the TNF-α group at day 10 contrasts with reports showing robust TNF-α-induced miR-155 upregulation at earlier time points. This discrepancy may reflect temporal dynamics in microRNA regulation, as miR-155 often exhibits transient induction during early inflammatory responses, with expression levels declining at later stages despite sustained functional inhibition of adipogenesis [56]. Thus, the lack of significant miR-155 differences at day 10 does not exclude its involvement in early anti-adipogenic signaling mediated by TNF-α. Taken together, these findings indicate that Aloe-emodin and TNF-α differentially regulate microRNAs involved in adipogenesis, with TNF-α exerting a strong and robust inhibitory effect consistent with inflammatory blockade, while emodin modulates microRNA expression in a condition-dependent manner. The dissociation between microRNA changes and terminal adipogenic gene expression observed in certain conditions highlights the complexity of post-transcriptional control during adipocyte differentiation and supports the view that emodin functions primarily as a regulatory or modulatory agent, rather than a direct inhibitor of adipogenesis.

Although much of the current literature suggests that Aloe-emodin exerts anti-adipogenic effects, the present study demonstrates, first, that the proposed concentration of 40 µM is associated with a metabolic shift that does not compromise cell viability, thereby allowing the differentiation process to proceed despite this modulatory effect. Furthermore, comparison with the group treated exclusively with Aloe-emodin in the absence of an adipogenic medium reveals that the compound alone does not possess the capacity to induce adipogenesis, yet it does modulate microRNA expression, as is visible in the Log_2_FC analysis. This opens up a new avenue of research to better understand the kinetics of Aloe-emodin and its relationship with the post-transcriptional regulation of microRNAs during the adipogenic process. Finally, while this study provides a robust transcriptomic and regulatory framework, future studies incorporating protein-level validation (Western blot for PPARγ or C/EBPβ) and functional assays will be essential to fully elucidate the precise translational dynamics of Aloe-emodin action in this cellular model.

## 5. Conclusions

In conclusion, these results demonstrate that Aloe-emodin exerts differential regulatory effects on microRNAs and transcription factors related to adipogenesis. Aloe-emodin at 40 µM is capable of modulating microRNA expression in a condition-dependent manner, playing a stronger role during the late stages of adipogenesis toward terminal differentiation rather than blocking lineage commitment; this suggests a modulated differentiation process, in which miR-143 actively participates. On the other hand, TNF-α acts as a potent inhibitor, consistent with transcriptional regulation of Pparg and Cebpb, as well as the upregulation of miR-155 expression. This highlights the complexity of post-transcriptional regulation during adipocyte differentiation and supports the role of Aloe-emodin as a potential modulating agent in differentiation rather than a strict anti-adipogenic agent. However, as a first exploratory step, further studies on the metabolic and gene expression kinetics are necessary to fully elucidate the exact pathways employed by Aloe- emodin in adipogenesis.

## Figures and Tables

**Table 1:** Primer sequences used for qPCR analysis.

Gene	Sequence		ID GenBank
	Forward	Reverse	
*Rplp0*	ATCGTCTTTAAACCCCGCGT	ACGTTGTCTGCTCCCACAAT	NM_007475.5
*Pparg1*	CTGTGAGACCAACAGCCTGA	AATGCGAGTGGTCTTCCATC	NM_001127330.3
*Cebpb*	TCGGGACTTGATGCAATCCG	TACACGTGTGTTGCGTCAGT	NM_001287738.1
*Fabp4*	TCACCTGGAAGACAGCTCCT	AATCCCCATTTACHCTGATG	NM_001409513.1
*Adipoq*	CCCAGTCATGCCGAAGATGA	CAAGTTCCCTTGGGTGGAGG	NM_009605.5
*Slc2a4* (*Glut4*)	AACACTAACCAACTGGCCA	CCAGCATAGACTCCAAGCCC	NM_001359114.2

**Table 2:** Sequences and assay IDs of miRNAs and U6 snRNA used for qPCR.

sn/miRNA	Sequence	ID Accession
Rnu6	GTGCTCGCTTCGGCAGCACATATACTAAAATTGGAACGATACAGAGAAGATTAGCATGGCCCCTGCGCAAGGATGACACGCAAATTCGTGAAGCGTTCCATATTTTT	NR_003027.2
Mir143	GGUGCAGUGCUGCAUCUCUGG	MI0000257
Mir155	UUAAUGCUAAUUGUGAUAGGGGU	MI0000177

## Data Availability

The raw data supporting the conclusions of this article will be made available by the authors on request.

## Supplementary Materials

The following supporting information can be downloaded at https://www.mdpi.com/article/10.3390/life16091552/s1.

## Abbreviations

The following abbreviations are used in this manuscript:

MSCs	Mesenchymal Stem Cells
PI3K	Phosphoinositide 3-Kinase
AKT	Protein Kinases
mTORC1	mechanistic Target of Rapamycin Complex 1
MAPK/ERK	Mitogen-Activated Protein Kinase/Extracellular signal-regulated kinase
ZFP423	Zinc Finger Factor 423
p38	Protein kinase 38
BMP2/4	Bone Morphogenetic Proteins 2 and 4
cAMP	cyclic Adenosine Monophosphate
CREB	Response Element-Binding Protein
PPARγ	Peroxisome Proliferator-Activated Receptor gamma
C/EBPα	Coactivator CCAAT/enhancer-binding protein alpha
C/EBPβ	Coactivator CCAAT/enhancer-binding protein beta
FABP4	Fatty Acid-Binding Protein 4
LPL	Lipoprotein Lipase
ADIPOQ	Adiponectin
GLUT4	Glucose Transporter 4
AT	Adipose tissue
TNF-α	Tumor Necrosis Factor-alpha
IL-6	Interleukin 6
IL-1β	Interleukin 1-beta
ROS	Reactive Oxygen Species
miRNAs	MicroRNAs
lncRNAs	long non-coding RNAs
CCL2	chemokine (C-C motif) ligand 2
PHB	Prohibitin
