# Supplementary material for: Aloe-Emodin Modifies Adipogenic Genes and the Expression of miR-143 and miR-155 in 3T3-L1 Cells
Source: Life (Basel). 2026 Sep 16;16(9):1552. doi: 10.3390/life16091552 (PMC13608205; doi:10.3390/life16091552)
Supplement: Supplementary file 1 [file life-16-01552-s001.zip › life-4486498-supplementary.pdf]

Supplementary material.

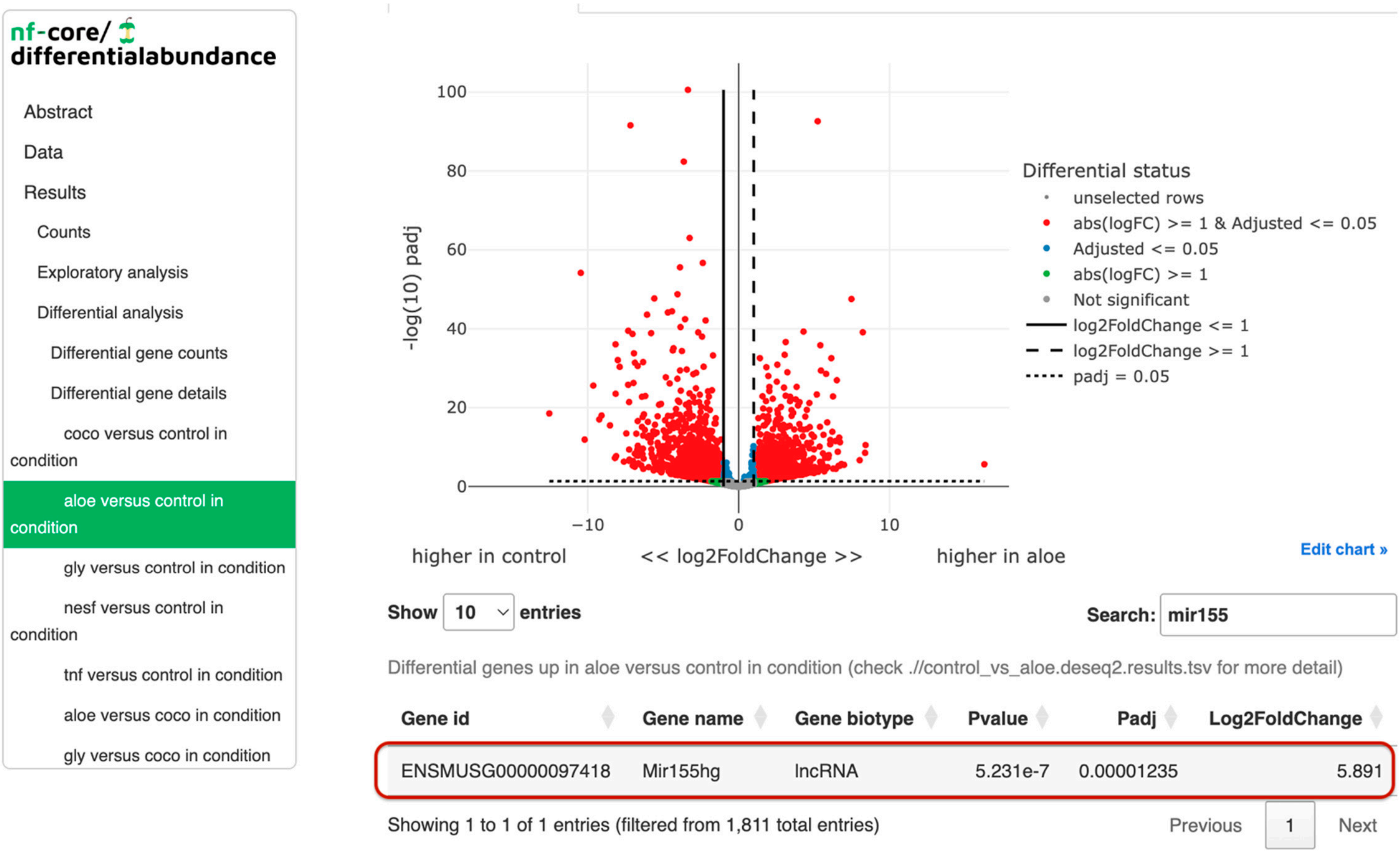

Figure S1. Volcano plate for Aloe-emodin treatment in 3T3-L1 cells. We can observe the $Log_2FoldChange$ value for Mir155hg (lncRNA), Pvalue and Padj.
